# The potential role of mesenchymal stem cells enhanced with melatonin in renal damage induced experimentally by cecal ligation and puncture in rats

**DOI:** 10.3389/fimmu.2026.1684692

**Published:** 2026-02-20

**Authors:** Amina F. Hussein, Manal F. El-Khadragy, Rasha S. Elbeltagy, Ahmed E. Abdel Moneim, Mohga S. Abdalla

**Affiliations:** 1 Chemistry Department, Biochemistry Division, Faculty of Science, Capital University, Cairo, Egypt; 2 Department of Biology, College of Science, Princess Nourah bint Abdulrahman University, Riyadh, Saudi Arabia; 3 Zoology and Entomology Department, Faculty of Science, Capital University, Cairo, Egypt; 4 Unit of Scientific Research, Applied College, Qassim University, Buraydah, Saudi Arabia

**Keywords:** acute kidney injury, melatonin, mesenchymal stem cells, multiorgan dysfunction, NF-κB pathway, sepsis

## Abstract

**Introduction:**

Sepsis-induced acute kidney injury (AKI) carries high mortality, and treatment options beyond supportive care are limited. Mesenchymal stem cells (MSCs) offer therapeutic potential due to their paracrine properties but are limited by poor survival and engraftment post-transplantation. Preconditioning with melatonin (MEL) may enhance MSC efficacy. This study aimed to compare the renoprotective effects of MSCs alone versus melatonin-preconditioned MSCs (MSCs+MEL) in a rat model of septic AKI.

**Methods:**

Polymicrobial sepsis was induced in male Wistar rats via cecal ligation and puncture (CLP). Three hours post-CLP, animals received an intraperitoneal injection of either MSCs (1 × 10^6), MSCs+MEL, or vehicle. Renal function, oxidative/antioxidant markers, inflammatory cytokines (IL-1β, IL-6, TNF-α, NF-κB), apoptosis, and histopathology were assessed.

**Results:**

CLP-induced sepsis resulted in significant AKI, evidenced by elevated inflammatory and apoptotic markers, oxidative stress, and histopathological damage. Both treatment groups showed improved kidney function and histology compared to untreated septic controls. However, the MSCs+MEL combination was significantly more effective. It superiorly reduced the expression of IL-1β, IL-6, TNF-α, NF-κB, attenuated oxidative stress and apoptosis, enhanced antioxidant defenses, and resulted in more pronounced histological improvement, as confirmed by immunohistochemistry.

**Discussion:**

Preconditioning with melatonin synergistically enhances the therapeutic efficacy of MSCs in septic AKI. The MSCs+MEL combination exerts superior renoprotection by more robustly mitigating inflammation, oxidative stress, and apoptosis while promoting tissue repair.

## Introduction

Sepsis is one of the leading causes of death worldwide, arising from syndromic reactions to several viral diseases. It is a potentially fatal clinical condition that arises from immune suppression caused by damage to the body’s tissues and organs in reaction to infection (1). Numerous morbidities, including liver, kidney, heart, and central nervous system dysfunction, are associated with sepsis (2). While the exact number of cases worldwide is unknown, 49 million instances of sepsis were estimated in 2017, and sepsis-related deaths accounted for 11 million deaths, or almost 20% of all causes of death globally (3). The fifth most frequent cause of acute kidney damage that is acquired in a hospital is sepsis-induced acute kidney injury (SI-AKI) (4). Prior research on patients in the intensive care unit revealed that between 20% and 30% of patients had sepsis, and nearly half of those patients had acute kidney injury (AKI). Additionally, SI-AKI is linked to a 76% increased risk of in-hospital mortality (5–8). Oliguria and decreased kidney solute clearance (9) are the clinical characteristics of SI-AKI, which typically lead to fluid overload, hazardous drug and metabolite buildup, and electrolyte and acid-base imbalances (10).

Significantly, the incidence rate of SI-AKI tends to increase in tandem with the worldwide aging trend (11). Additionally, the older population is particularly susceptible to sepsis. Treatment for SI-AKI is unsatisfactory; neither supportive therapy nor a particular medication against SI-AKI is clinically accessible (12). As chronic inflammation plays a significant role in the pathophysiology of sepsis, reducing inflammation may be a useful strategy for averting organ failure. Because of their capacity for tissue regeneration and anti-inflammatory properties (13), mesenchymal stem cells (MSCs) have gained interest in the treatment of sepsis. The therapeutic potential of MSCs to regulate inflammation and lessen tissue damage brought on by sepsis has been the subject of numerous investigations. Bone marrow–derived mesenchymal stem cells (BMSCs) are essential for tissue maintenance, repair, and regeneration because of their capacity for self-renewal and multidifferentiation (14).

For AKI, BMSCs have been shown to have beneficial therapeutic benefits (15). With the benefits of minimal toxicity and auto transplantation, BMSCs have a wide range of potential applications in the clinical treatment of AKI. They can localize to kidney and stimulate kidney regeneration by differentiation or paracrine activity (16). In rats with sepsis caused by cecal ligation and puncture (CLP), Xu et al. have demonstrated that BMSC injection can lower mortality, alleviate lung injury, and decrease levels of pro-inflammatory markers (17). Unfortunately, there is a chance that these elements will have negative consequences and be harmful (18). Consequently, a crucial tactic to maximize BMSCs’ potential for application in cell-based therapy is to combine them with natural chemical products (19, 20). One of these products is melatonin (MEL). MEL is a neurohormone secreted by the pineal gland, which has several uses, including regulating circadian rhythms, reducing inflammation, and reducing oxidative stress (21, 22). It has been shown that using MEL in MSCs preconditioning improved the therapeutic result in AKI models. The underlying mechanism of MEL’s ability to inhibit the formation of reactive oxygen species (ROS) and oxidative stress may be due to its ability to act either receptor-independently or receptor-dependently through the AMPK/ACC, PrPC/PINK1, and ERK1/2 signaling pathways (23, 24). The purpose of this study is to examine the protective properties of stem cells on their own and in combination with MEL against oxidative stress and inflammation brought on by sepsis induction in rats.

## Materials & methods

### Chemicals, experimental groups, and CLP model

#### Chemicals

A 10% povidone-iodine solution (Betadine; Alcon Pharma, Egypt) was used for disinfection, and ketamine (Ketalar^®^, Hospira, Egypt) and xylazine hydrochloride (Rompun^®^, Sigma-Aldrich, Egypt) were used for anesthesia. For bone marrow isolation and culture, a 10% fetal bovine serum (FBS, Gibco, Fisher Scientific, Cat. No. 16000044), 100 IU/ml streptomycin, and a combination of 100 IU/ml penicillin (Dulbecco’s Modified Eagle Medium, Sigma, St. Louis, MO, USA) were used. The BMSCs were pretreated with 100 µM MEL (CAS number 73–31–4; Sigma, St. Louis, MO, USA), and a 0.25% trypsin solution (Sigma, St. Louis, MO, USA, product no. PP0100) was used to extract adherent cells. Serum blood urea nitrogen (BUN, Cat. No. MAK006), CRP (C-reactive protein, Cat. No. E-EL-H0043), and creatinine (Creat, Cat. No. MAK080) were evaluated using a colorimetric technique (Sigma-Aldrich CO.). The ELISA kits for measuring apoptotic proteins (Bax, caspase-3, and Bcl2) were purchased from Cusabio, Elab Science, and LS Bio companies, respectively. Kits for TNF-α and IL-1β were purchased from Thermo Fisher Scientific and Ray Biotech. ELISA kits for measuring Kim-1, NF-κB, IL-6, PGE2, and COX2 were purchased from Elab Science. The kit for total RNA extraction was purchased from Thermo Fisher Scientific (TRIzol reagent and RevertAidTM H Minus Reverse Transcriptase). The QuantiFast SYBR Green RT-PCR kit was purchased from Qiagen, CO, Germany. The Thermo Fisher Scientific ViiATM 7 System was used to perform duplicates of each reaction. The housekeeping gene utilized was glyceraldehyde-3-phosphate dehydrogenase (Gapdh), and the primers for CASPS 3, MAPK 9, and INOS genes were purchased from Jena Bioscience, Jena, Germany.

#### Experimental animals

Male Wistar albino rats were purchased from VACSERA, Giza, Egypt. They weighed between 180 and 220 g and were 10 weeks old on average. They were kept in wire polypropylene cages with a 12h light/dark cycle in controlled conditions at a temperature of 25 ± 2°C and a relative humidity of 55% ± 15%. Every rat received a regular diet along with an unrestricted supply of water. Animal care and use followed the guidelines of Investigations & Ethics for Laboratory Animal Care at the Faculty of Science, Capital University (Approval No. HU-IACUC/Z/AEA0221-2).

#### Induction of sepsis by the cecal ligation and puncture (CLP) model

A longitudinal incision was made by scalpel beneath the xiphoid cartilage of rats, and then, using an 18-gauge needle, a single pass was made through the cecum to puncture it. The needle was removed, and a small amount of feces was extruded to make sure of the holes. The cecum was replaced in the abdominal cavity, and the incision was stitched using 3.0 silk sutures. To provide postoperative fluid resuscitation, we subcutaneously injected normal saline (0.9% NaCl, 50 ml/kg, 37 °C). After the operation, we returned the rats to their cages. The animals had unrestricted access to water and food (25).

#### Bone marrow–derived MSCs (isolation and culture)

Bone marrow–derived MSCs were isolated from three male albino Wistar rats (average age: 10 weeks). Following deep sedation via intraperitoneal injection of pentobarbital (300 mg/kg), euthanasia was performed by cervical dislocation. The tibias and femurs were aseptically excised, and all adherent muscle and connective tissues were meticulously removed. Both epiphyses were cut, and the marrow cavity was flushed repeatedly using a 3-ml syringe and a 21-gauge needle filled with complete culture medium (α-MEM supplemented with 10% fetal bovine serum, 100 IU/ml penicillin, and 100 µg/ml streptomycin) until the bone appeared pale. The resulting bone marrow suspension was passed through a 70-µm cell strainer to obtain a single-cell suspension. Cells were plated in T-75 flasks and maintained at 37 °C in a humidified 5% CO_2_ incubator. After 24h, non-adherent cells were removed by a complete medium change. The medium was subsequently replaced every three days. Adherent, spindle-shaped MSCs were expanded until passage 3 for experimental use according to Wan et al. (26). When the BMSCs reached 80% confluency, they were pretreated with 100 µM MEL. The MEL solution was freshly prepared in a vehicle of 99% saline and 1% pure ethanol (96%), sterilized by filtration through a 0.22 µm syringe filter (PES membrane), and then applied to the cells. Twenty-four hours after MEL treatment, adherent cells were detached for *in-vivo* investigations using a 0.25% trypsin solution.

Animal groups:

Rats were divided into five groups as follows:

1. Control group: received only oral saline solution (27).
2. Sham group: A longitudinal incision was made at a distance of 2–2.5 cm beneath the xiphoid cartilage of rats. The abdominal cavities of animals were opened and closed back again without performing the CLP procedure.
3. Sepsis (vehicle) group: A CLP model was created according to Rittirsch et al. and Song et al. (28, 29). Three hours following the CLP procedure, rats received an injection i.p. of 0.2 ml normal saline.
4. MSCs group: Three hours following the CLP procedure, rats received an injection i.p. of 0.2 ml normal saline containing 1 × 10^6 BMSCs; BMSCs were first labeled on ice with 4 mg/L chloromethyl benzamide dialkylcarbocyanine (CM-Dil) (Thermo Fisher Scientific, Cat. No.: C7001) for 15 min prior to injection (30).
5. MSCs + MEL group: Rats received an i.p. injection of 1 × 10^6 BMSCs that had been preconditioned with 100 µM melatonin for 24h prior to harvesting (31).

### Methods

#### Collection of samples

Animals were anesthetized and euthanized by cervical dislocation at 48h after the CLP procedure, followed by dissection and collection of the renal tissues. To obtain the serum, blood samples were collected and incubated for an hour at 4 °C, then were centrifuged for 10 min at 3,000*g*. The kidney tissues were quickly removed and split into three parts. For histological examination, the first portion was fixed at 25 °C in 10% neutral formalin. The second portion was kept at −80 °C for RNA isolation to analyze gene expression. The third portion was homogenized with phosphate buffer, then centrifuged (9,000–10,000 rpm), and the supernatant was kept at −70 °C for biochemical analysis, oxidative stress markers, and ELISA technique (32).

#### Biochemical measurements

Serum blood urea nitrogen and creatinine were evaluated using a colorimetric technique according to the manufacturer’s protocol. ELISA kits for CRP were used to quantify its serum levels in accordance with the manufacturer’s instructions.

#### Oxidative stress and antioxidant markers

Using the method of Ohkawa et al. (33), kidney homogenate was used to measure malondialdehyde (MDA) as an indication of lipid peroxidation (LPO) by reaction of thiobarbituric acid. For glutathione (GSH) (34) and nitrite/nitrate (nitric oxide; NO) (35) were also chemically measured. Superoxide dismutase (SOD) activity was measured in kidney supernatants to assess the enzymatic antioxidant state, in accordance with Nishikimi et al. (36). The catalase (CAT) activity was tested according to Aebi (37). The activities of glutathione reductase (GR) and glutathione peroxidase (GPx) were measured using the techniques of Paglia and Valentine (38) and Carlberg and Mannervik (39), respectively. Myeloperoxidase (MPO) activity was measured using the Bradley et al. (40) methodology.

#### Inflammatory markers

ELISA kits were used to determine the levels of apoptotic proteins (Bax, caspase-3, and Bcl-2) in kidney homogenates from all experimental groups. The catalog numbers for Bax, caspase-3, and Bcl-2 ELISA kits were CSB-EL002573RA, E-EL-R0160, and LS-F4135, respectively. ELISA kits were also used to measure the concentrations of TNF-α (Thermo Fisher Scientific, Cat. No. MBS2507393) and IL-1β (RayBiotech, Cat. No. ELR-IL-1β) in kidney homogenates, as well as NF-κB DNA-binding activity in the nuclear fraction, which was assessed using an ELISA kit from Elabscience (Cat. No. E-EL-R0674), and the activity was expressed as NF-κB p65 protein (ng/mg). The concentration of IL-6 was quantified using a commercial ELISA kit (Cat. No. MBS269892). In addition, PGE2 concentration (Elabscience, Cat. No. E-EL-0034) and COX-2 levels (Cat. No. MBS266603) were measured using ELISA kits. All procedures were performed according to the manufacturers’ instructions. The plasma level of Kim-1 was measured using the Elabscience ELISA kit (Cat. No. E-EL-R3019).

#### Gene expression

Total RNA was extracted from renal tissue using TRIzol reagent, and then RevertAidTM H Minus Reverse Transcriptase (Fermentas, Thermo Fisher Scientific, Cat. No. EP0451) was used to synthesize cDNA in accordance with the manufacturer’s procedure. The QuantiFast SYBR Green RT-PCR kit (Qiagen, Cat. No. 204156) was utilized for qRT-PCR. Thermo Fisher Scientific (Cat. No. 4453546) ViiATM 7 System was used to perform duplicates of each reaction. Denaturation at 95 °C for 12 min was the first step in PCR cycling conditions. This was followed by 40 cycles of denaturation at 94 °C for 60 s, annealing at 58 °C for 60 s, extension at 72 °C for 90 s, and holding for a final extension at 72 °C for 10 min. The ΔΔCt method was used to determine the difference in the mean expression levels of the studied genes, normalized to the expression of glyceraldehyde 3-phosphate dehydrogenase (GAPDH). According to Abdel Moneim (41). **Table 1** lists the primers for estimating the expressions of *CASP 3*, *MAPK 9*, and *INOS* genes.

**Table 1:** The primers sequences and accession numbers of genes analyzed in real-time PCR.

Name	Accession no.	Sense (5′-3′)	Antisense (3′–5′)
*GAPDH*	NM_017008.4	AGACAGCCGCATCTTCTTGT	TACGGCCAAATCCGTTCACA
*CASP 3*	NM_012922.2	GAGCTTGGAACGCGAAGAAA	TTGCGAGCTGACATTCCAGT
*INOS 2*	NM_001429940.1	CCAGCTATGCGCGAGGG	GTGGTGAAGGGTGTCGTGAA
*MAPK 9*	NM_017322.2	CGGACAGCCTGTACCAACTT	CCAGCTCTCCCATGATGCAA

#### Histological examinations

Samples of kidney tissues were preserved in 10% formaldehyde for at least 24h, then subjected to standard procedures for paraffin embedding. After that, the tissues were cut into 3 μm slices and stained using hematoxylin-eosin (H&E) reagent for light microscopy analysis (42). A semi-quantitative assessment was given to each degree of pathological change. The pathological alterations under investigation include inflammatory cell infiltration, glomerular degeneration, vacuolar degeneration, edema, and/or cellular debris.

#### Immunohistochemical studies

An immunohistochemical procedure using a monoclonal anti–NF-κB antibody (Sigma-Aldrich, Egypt) (Product No.: N8523) was used to examine nuclear factor kappa B (NF-κB), and a TNF alpha polyclonal antibody (Thermo Fisher, Egypt) (Cat No: PA1-40281) to examine TNF-α in kidney tissue according to the manufacturer’s protocol. A 400× magnification lens (Nikon Eclipse E200-LED, Helwan, Egypt) was used to examine kidney slices (43). The immunohistochemical staining for NF-κB and TNF-α was quantitatively analyzed using digital image analysis. Image analysis was performed using ImageJ software (National Institutes of Health, USA). Color images were converted to an appropriate color space, and the brown diaminobenzidine (DAB) signal was isolated using standardized threshold settings. The integrated optical density (IOD) and mean gray value were measured, and background staining was subtracted from each measurement. The average value of all fields per animal was calculated and used for statistical analysis.

#### Statistical analysis

For all values, the mean ± standard deviation (SD) was used. Quantitative immunohistochemical analysis was performed using ImageJ software. Multiple non-overlapping of five fields per section were analyzed. One-way analysis of variance (ANOVA) followed by Tukey’s multiple comparison test was used to analyze the collected data. Data were expressed as mean ± SD. A *p*-value < 0.05 was considered statistically significant.

## Results

### Melatonin-enhanced MSCs ameliorate systemic and renal dysfunction in CLP-induced kidney dysfunction

**Figure 1:**
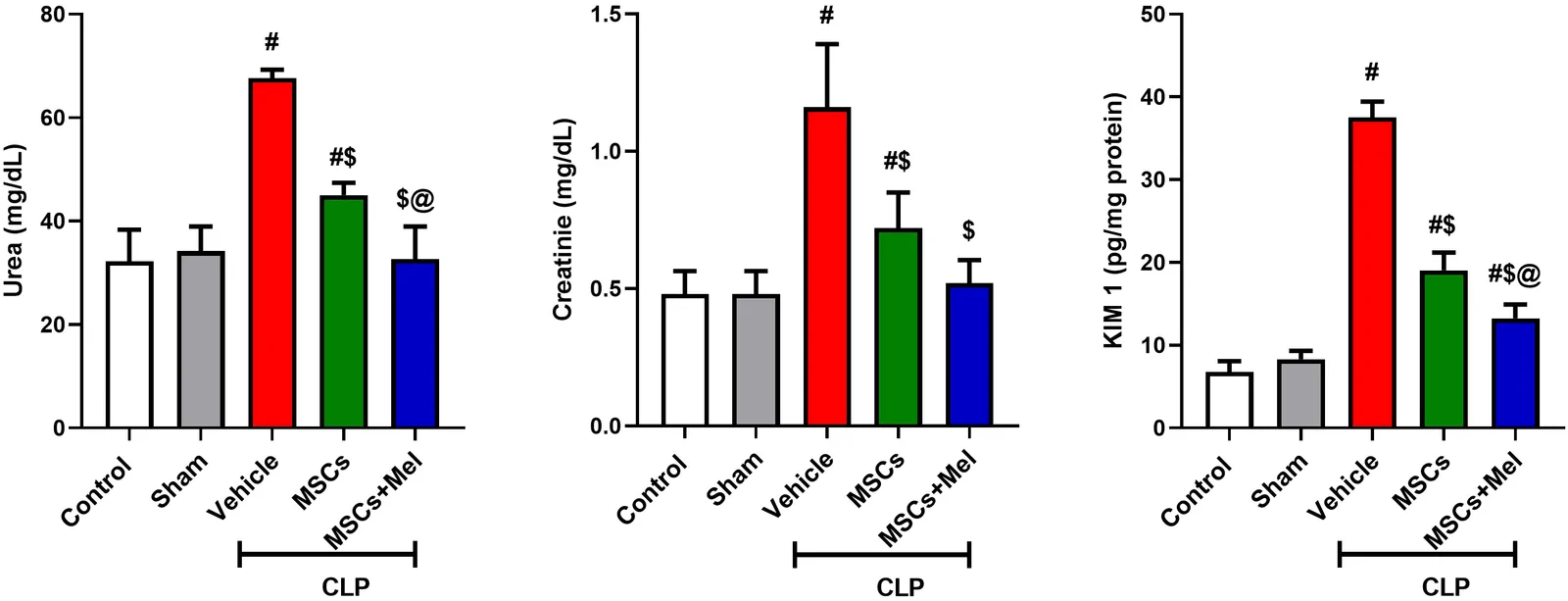
The renoprotective effect of mesenchymal stem cells preconditioned with melatonin on serum renal function parameters, namely, BUN, creatinine, and KIM-1, in septic rats after cecal ligation and puncture. Data displayed as mean ± SD (*n* = 5). Statistical comparisons among groups were performed using one-way analysis of variance (ANOVA) followed by Tukey’s *post-hoc* multiple comparison test. ^#^Differs from the control group (*P* < 0.05). ^$^Differs from the septic rats (*P* < 0.05). ^@^Differs from the CLP-MSCs rats (*P* < 0.05).

As shown in **Figure 1**, the septic rats’ renal function parameters (BUN, creatinine, and KIM-1) were noticeably higher than those of the control rats. Nevertheless, these CLP-triggered changes were considerably reduced in the MSCs group and the MSCs + MEL group. The two groups, MSCs and MSCs + MEL, showed a marked decrease in BUN and KIM-1 levels.

### Melatonin-enhanced MSCs attenuate renal oxidative stress and potentiate the antioxidant system

**Figure 2:**
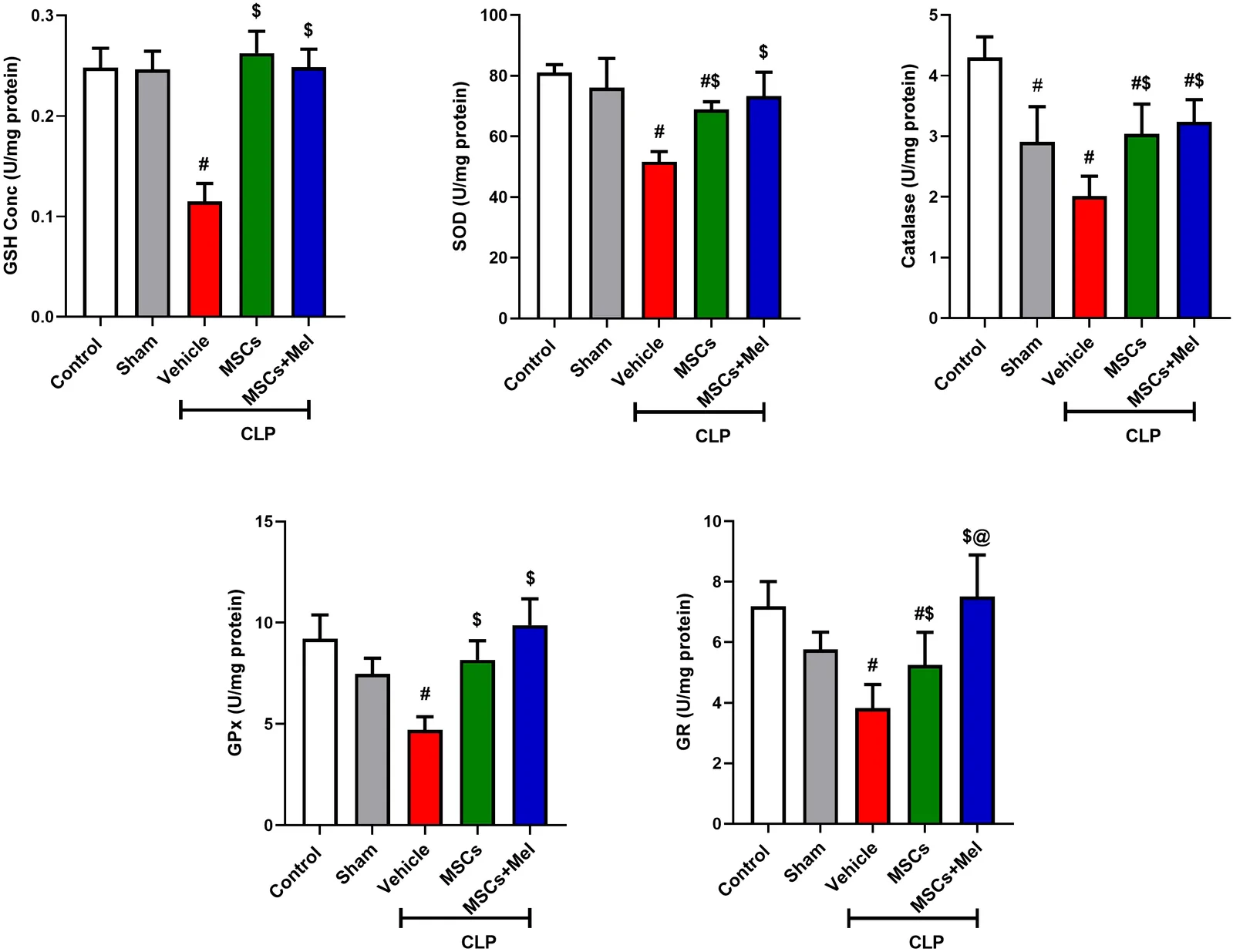
The renoprotective effect of mesenchymal stem cells preconditioned with melatonin on the antioxidant levels of SOD, CAT, GPx, GR, and GSH in renal tissue in septic rats after cecal ligation and puncture. Data displayed as mean ± SD (*n* = 5). Statistical comparisons among groups were performed using one-way analysis of variance (ANOVA) followed by Tukey’s *post-hoc* multiple comparison test. ^#^Differs from the control group (*P* < 0.05). ^$^Differs from the septic rats (*P* < 0.05). ^@^Differs from the CLP-MSCs rats (*P* < 0.05).

Oxidative stress was assessed in renal tissues 48h post-CLP. As shown in **Figure 2**, sepsis induction resulted in a significant depletion of the antioxidant defense system compared to control and sham groups. The levels of reduced glutathione (GSH) and the enzymatic activities of superoxide dismutase (SOD), catalase (CAT), glutathione peroxidase (GPx), and glutathione reductase (GR) were markedly lower in the sepsis (CLP) group (*P* < 0.05). Treatment with MSCs alone significantly restored the activities of these antioxidant markers. Notably, the combined treatment with MEL-preconditioned MSCs (MSCs + MEL) produced a more pronounced restoration, elevating GSH, SOD, CAT, GPx, and GR levels significantly higher than those in the sepsis group and, in several cases, surpassing the levels achieved with MSCs alone (*P* < 0.05).

**Figure 3:**
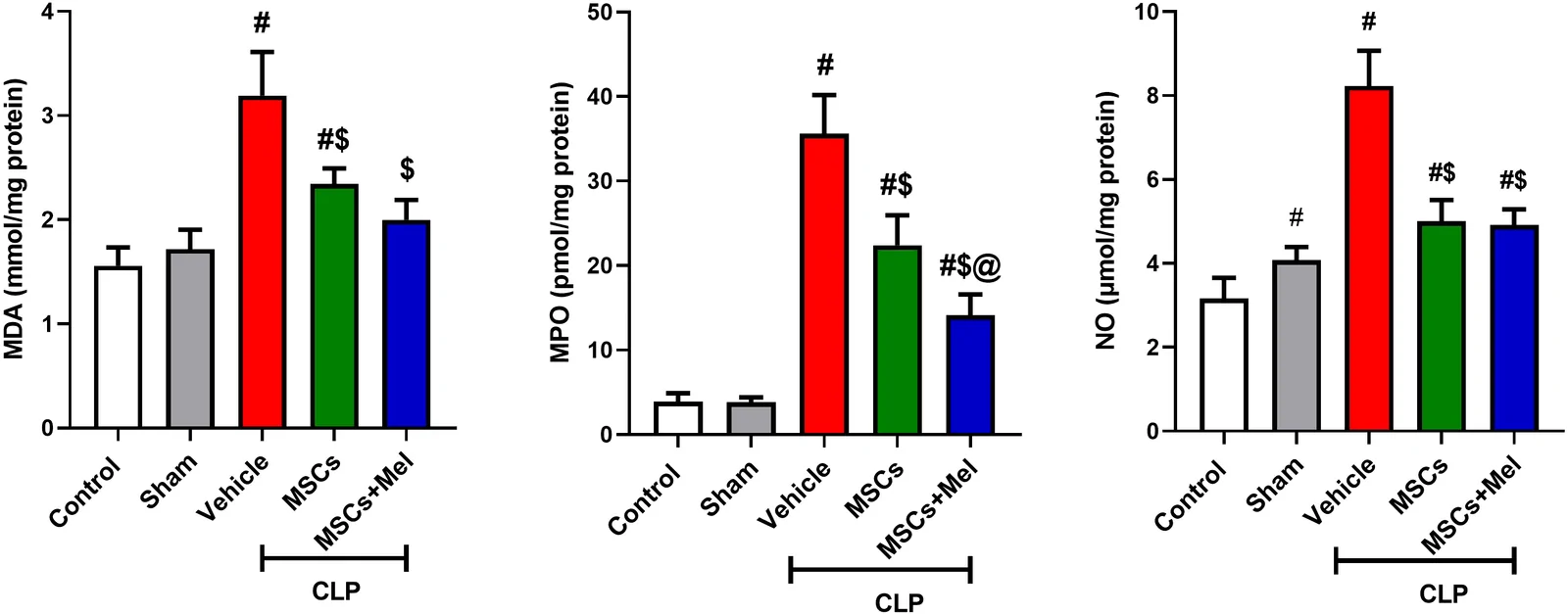
The renoprotective effect of mesenchymal stem cells preconditioned with melatonin on the oxidative stress markers (MDA, MPO, and NO) in renal tissue in septic rats after cecal ligation and puncture. Data displayed as mean ± SD (*n* = 5). Statistical comparisons among groups were performed using one-way analysis of variance (ANOVA) followed by Tukey’s *post-hoc* multiple comparison test. ^#^Differs from the control group (*P* < 0.05). ^$^Differs from the septic rats (*P* < 0.05). ^@^Differs from the CLP-MSCs rats (*P* < 0.05).

The impact of treatments on markers of oxidative damage is presented in **Figure 3**. Induction of sepsis led to a significant increase in lipid peroxidation, as evidenced by elevated malondialdehyde (MDA) levels, alongside increased myeloperoxidase (MPO) activity and nitric oxide (NO) concentration in renal tissue compared to control groups (*P* < 0.05). Both therapeutic interventions—MSCs and MSCs + MEL—effectively counteracted this oxidative damage. Administration of MSCs alone significantly reduced MDA, MPO, and NO levels compared to the untreated sepsis group. The renoprotective effect was further amplified in the MSCs + MEL group, which showed the most substantial reduction in all three oxidative damage markers, achieving levels significantly lower than those in the sepsis group and, for MDA and NO, significantly lower than in the group receiving MSCs alone (*P* < 0.05).

### Melatonin-enhanced MSCs restrain renal inflammation

**Figure 4:**
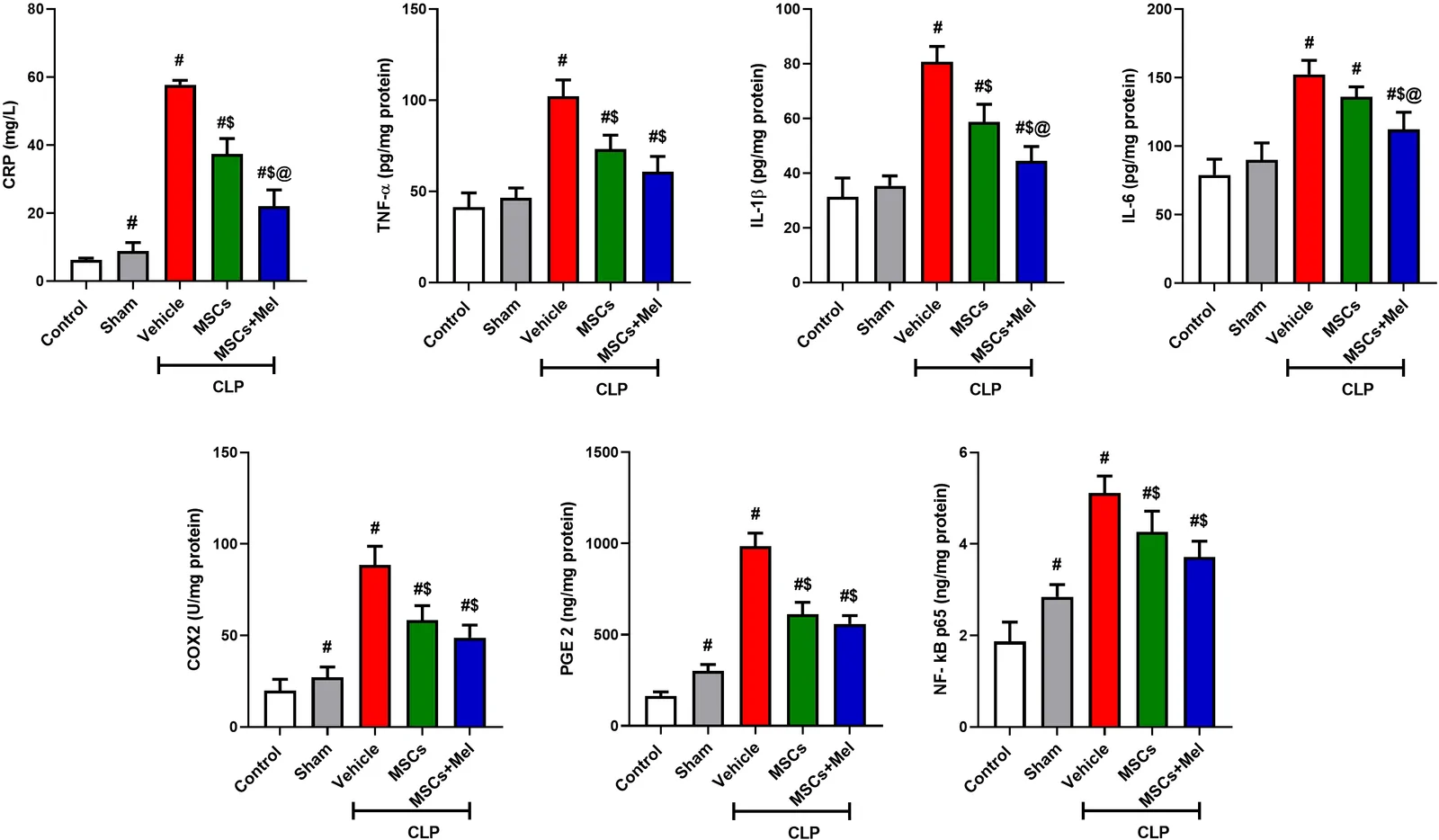
The renoprotective effect of mesenchymal stem cells preconditioned with melatonin on the pro-inflammatory markers (CRP, TNF-α, IL-1β, IL-6, COX2, PGE2, and NF-κB) in renal tissue in septic rats after cecal ligation and puncture. Data displayed as mean ± SD (*n* = 5). Statistical comparisons among groups were performed using one-way analysis of variance (ANOVA) followed by Tukey’s *post-hoc* multiple comparison test. ^#^Differs from the control group (*P* < 0.05). ^$^Differs from the septic rats (*P* < 0.05). ^@^Differs from the CLP-MSCs rats (*P* < 0.05).

The effect of treatments on key inflammatory mediators in renal tissue is presented in **Figure 4**. Induction of sepsis via CLP provoked a robust inflammatory response, resulting in significantly elevated levels of C-reactive protein (CRP) and the pro-inflammatory cytokines tumor necrosis factor-alpha (TNF-α), interleukin-1 beta (IL-1β), and interleukin-6 (IL-6), as well as increased levels of cyclooxygenase-2 (COX-2), prostaglandin E2 (PGE2), and nuclear factor kappa-B (NF-κB) compared to the control and sham groups (*P* < 0.05).

Both therapeutic interventions effectively mitigated this inflammatory cascade. Administration of MSCs alone significantly reduced the levels of all measured inflammatory markers compared to the untreated sepsis group. The anti-inflammatory effect was significantly enhanced in the group receiving MEL-preconditioned MSCs (MSCs + MEL). This combined treatment resulted in the most substantial suppression of CRP, TNF-α, IL-1β, IL-6, COX-2, PGE2, and NF-κB, with levels for several markers being significantly lower than those in the group treated with MSCs alone (*P* < 0.05).

**Figure 5:**
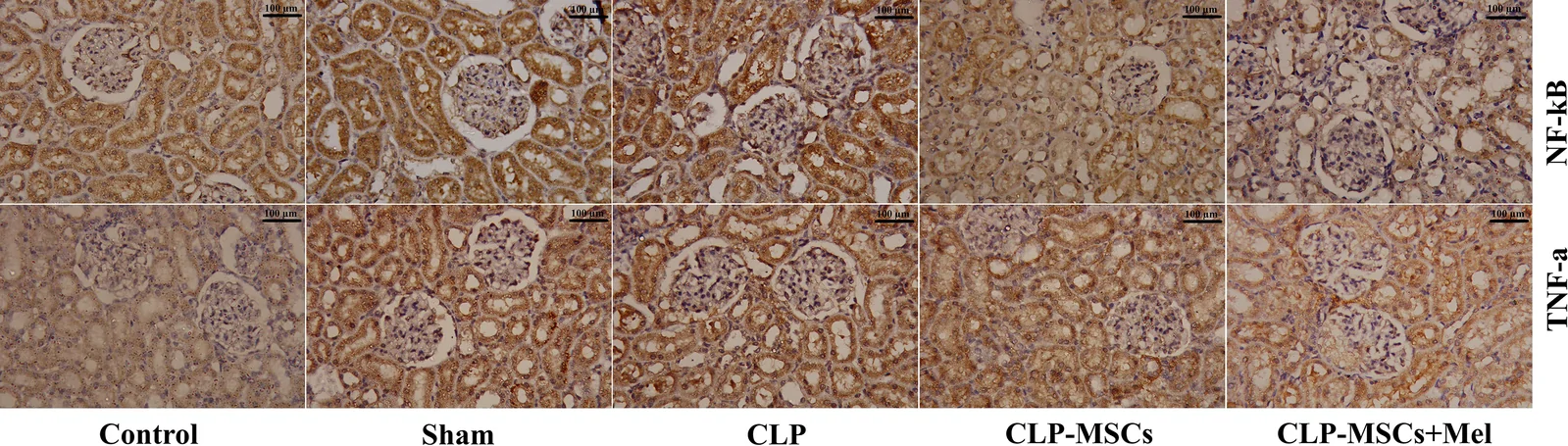
Shows immunohistological examinations of kidney sections that were taken from control, sham, sepsis, MSCs, and MSCs+MEL-treated rats. Demonstrating differences between the sepsis group’s TNF-α and NF-κB expressions and those of the MSCs and MSCs + MEL-treated groups. TNF-α and NF-κB expressions were lower in the MSCs and MSCs + MEL groups than in the sepsis group (×400; scale bar =100 µm).

**Figure 6:**
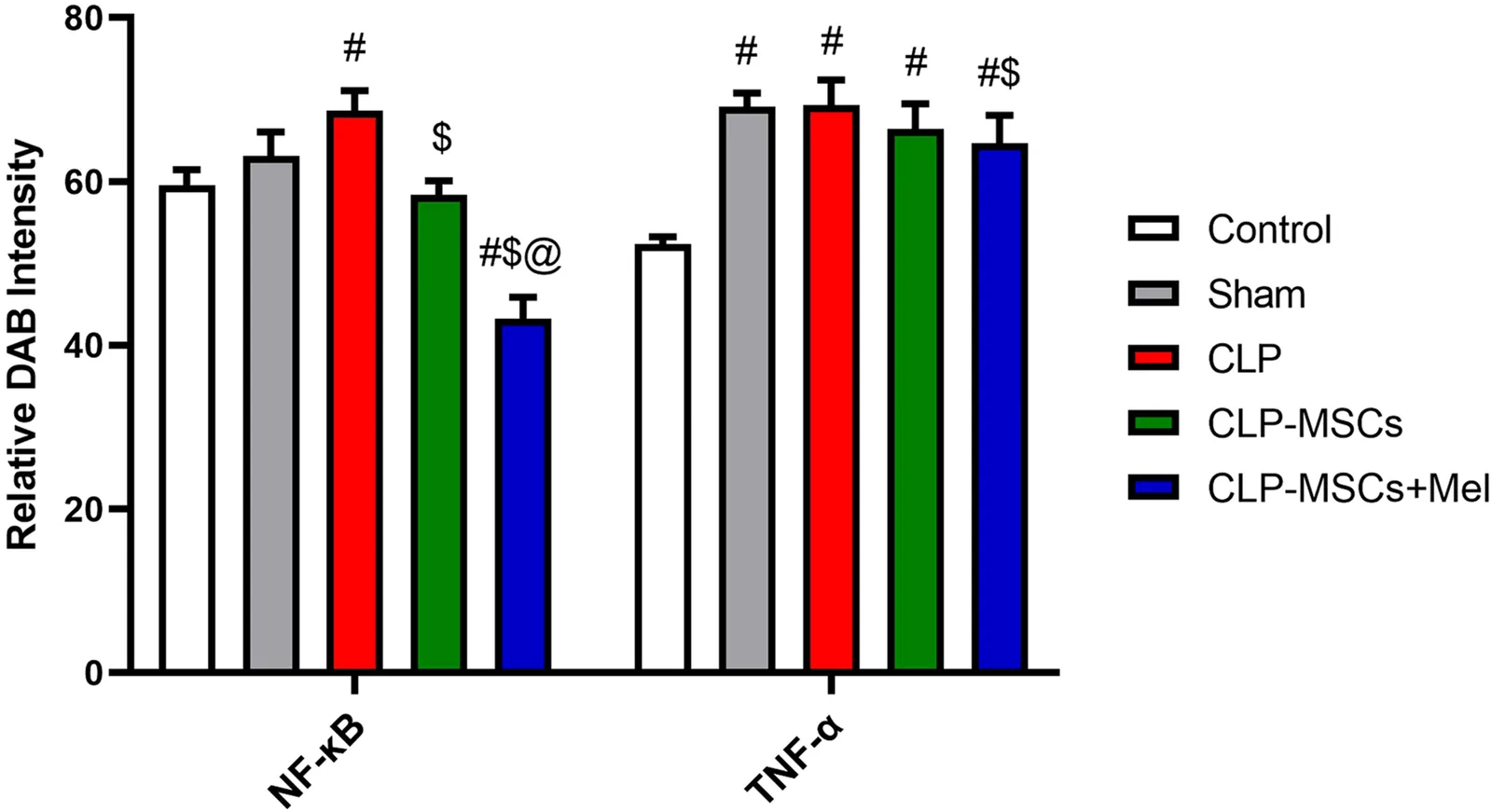
Quantitative analysis of diaminobenzidine (DAB) staining intensity for NF-κB and TNF-α in renal tissue across the Control, Sham, CLP, CLP-MSCs, and CLP-MSCs + MEL groups. DAB intensity was quantified using digital image analysis and expressed as mean ± SD (*n* = 5). Statistical comparisons among groups were performed using one-way analysis of variance (ANOVA) followed by Tukey’s *post-hoc* multiple comparison test. ^#^Differs from the control group (*P* < 0.05). ^$^Differs from the septic rats (*P* < 0.05). ^@^Differs from the CLP-MSCs rats (*P* < 0.05).

The anti-inflammatory effects observed at the protein level (**Figure 4**) were corroborated and localized by immunohistochemical analysis of NF-κB and TNF-α in renal tissues (**Figure 5**). Quantitative digital analysis of diaminobenzidine (DAB) staining intensity is presented in **Figure 6**. Induction of sepsis (CLP group) led to a significant increase in the nuclear expression of NF-κB and the cytoplasmic expression of TNF-α compared to control and sham groups (*P* < 0.05), indicating activation of pro-inflammatory pathways.

Treatment with MSCs alone (CLP-MSCs group) significantly attenuated this response, reducing the immunoreactivity of both NF-κB and TNF-α. Notably, the combined therapy with MEL-preconditioned MSCs (CLP-MSCs + MEL group) produced the most potent inhibitory effect. This group exhibited the lowest levels of NF-κB and TNF-α expression, which were significantly reduced compared to both the untreated sepsis (CLP) group and the group treated with MSCs alone (*P* < 0.05). This quantitative data visually supports the synergistic action of MEL in enhancing the anti-inflammatory capacity of MSCs, primarily through potent inhibition of the NF-κB pathway.

### Melatonin-enhanced MSCs attenuate renal apoptosis

**Figure 7:**
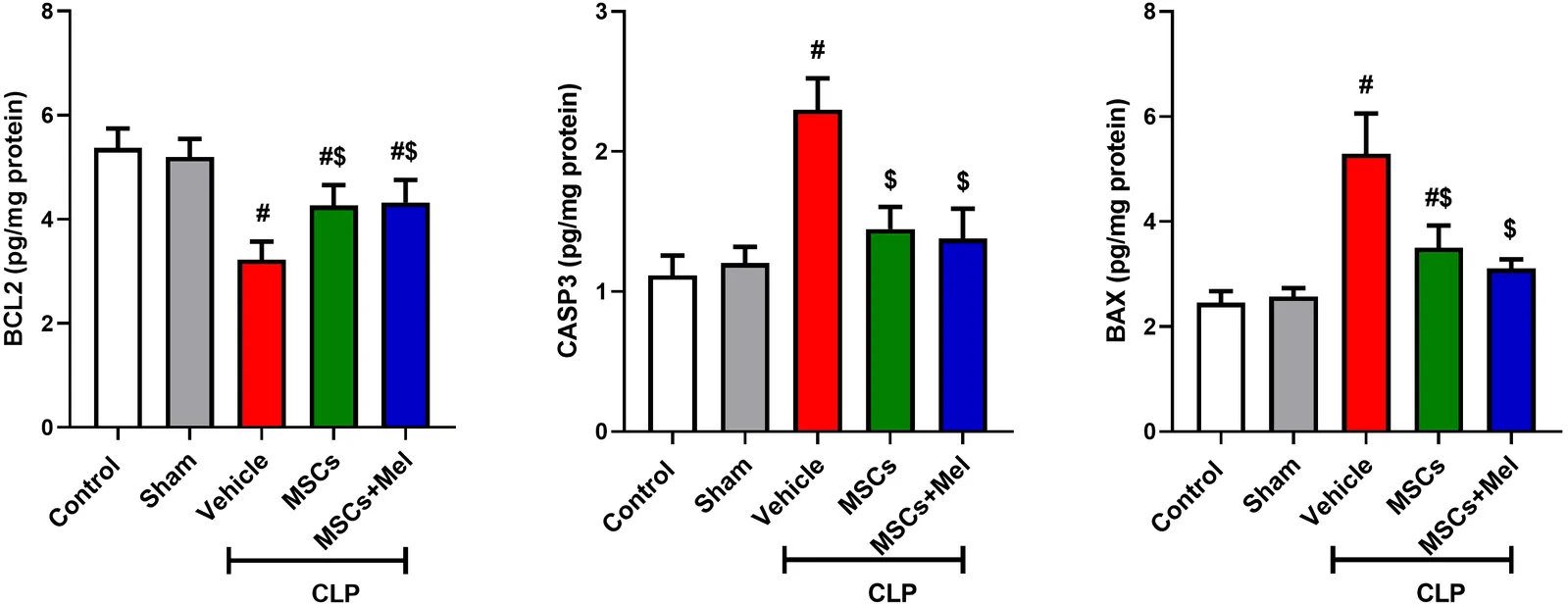
The renoprotective effect of mesenchymal stem cells preconditioned with melatonin on the apoptotic markers (Bcl2, Casp3, and Bax) in renal tissue in septic rats after cecal ligation and puncture. Data displayed as mean ± SD (*n* = 5). Statistical comparisons among groups were performed using one-way analysis of variance (ANOVA) followed by Tukey’s *post-hoc* multiple comparison test. ^#^Differs from the control group (*P* < 0.05). ^$^Differs from the septic rats (*P* < 0.05). ^@^Differs from the CLP-MSCs rats (*P* < 0.05).

**Figure 7** shows the markers linked to apoptosis in the renal tissue of various groups. In the sepsis group, there were noticeable decreases (*P* < 0.05) in the levels of the anti-apoptotic marker (Bcl-2) and increases (*P* < 0.05) in the levels of pro-apoptotic indicators (Casp-3 and Bax). However, the sepsis group that was treated with MSCs or MSCs + MEL had higher Bcl-2 levels and significantly lower (*P* < 0.05) Casp-3 and Bax levels.

**Figure 8:**
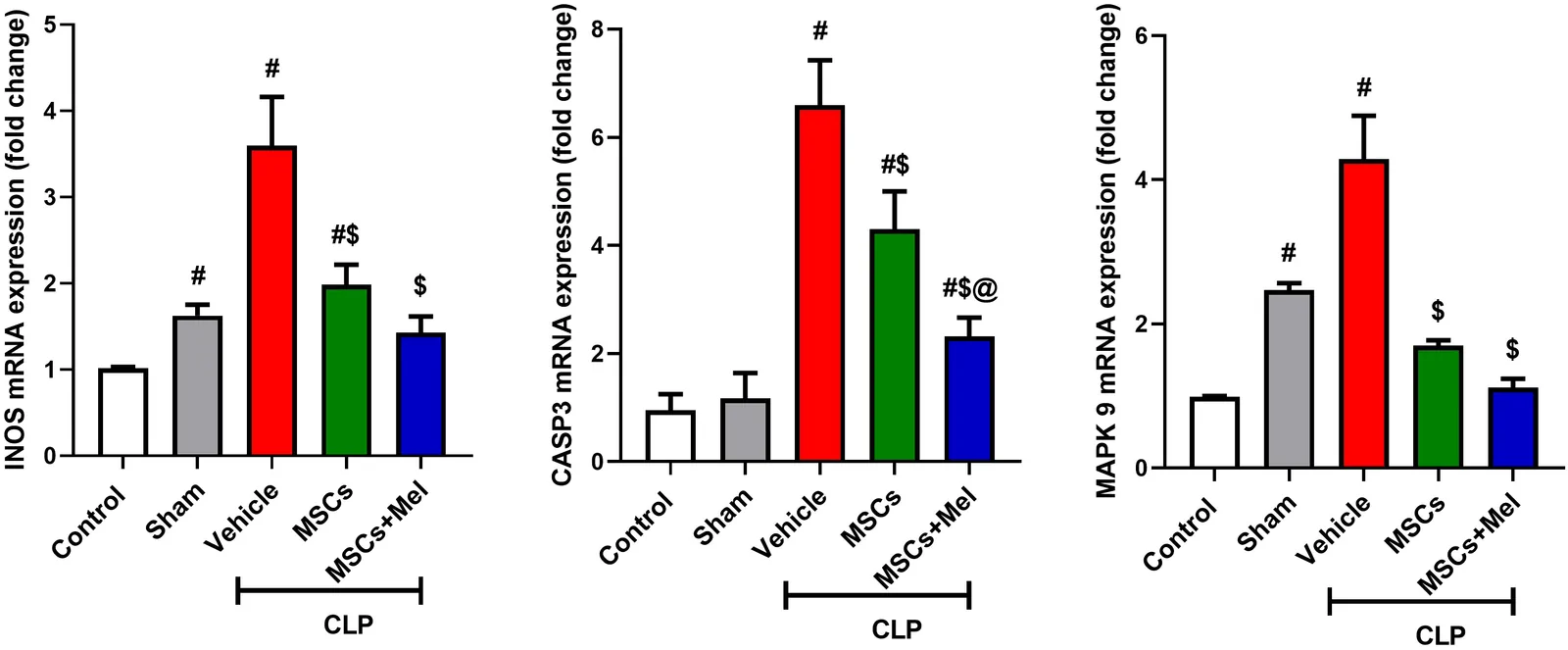
The renoprotective effect of mesenchymal stem cells preconditioned with melatonin on the gene expression levels of INOS, CASP 3, and MAPK 9 in renal tissue in septic rats after cecal ligation and puncture. Data displayed as mean ± SD (*n* = 5). Statistical comparisons among groups were performed using one-way analysis of variance (ANOVA) followed by Tukey’s *post-hoc* multiple comparison test. ^#^Differs from the control group (*P* < 0.05). ^$^Differs from the septic rats (*P* < 0.05). ^@^Differs from the CLP-MSCs rats (*P* < 0.05).

The impact of treatments on the transcriptional regulation of key genes involved in inflammation and apoptosis was assessed by qRT-PCR (**Figure 8**). Induction of sepsis (CLP group) significantly upregulated the mRNA expression of inducible nitric oxide synthase (*INOS*), cysteinyl aspartate-specific protease-3 (*CASP3*), and mitogen-activated protein kinase 9 (*MAPK9*) in renal tissue compared to control and sham groups (*P* < 0.05). Therapeutic intervention with MSCs alone (CLP-MSCs group) effectively countered this upregulation, significantly reducing the expression levels of all three genes. This suppressive effect was markedly potentiated in the group receiving MEL-preconditioned MSCs (CLP-MSCs + MEL group). The combined treatment resulted in the most substantial downregulation of *INOS*, *CASP3*, and *MAPK9* mRNA, with expression levels significantly lower than those in both the untreated sepsis group and the group treated with MSCs alone (*P* < 0.05).

### Melatonin-enhanced MSCs attenuate renal histopathological injury

**Figure 9:**
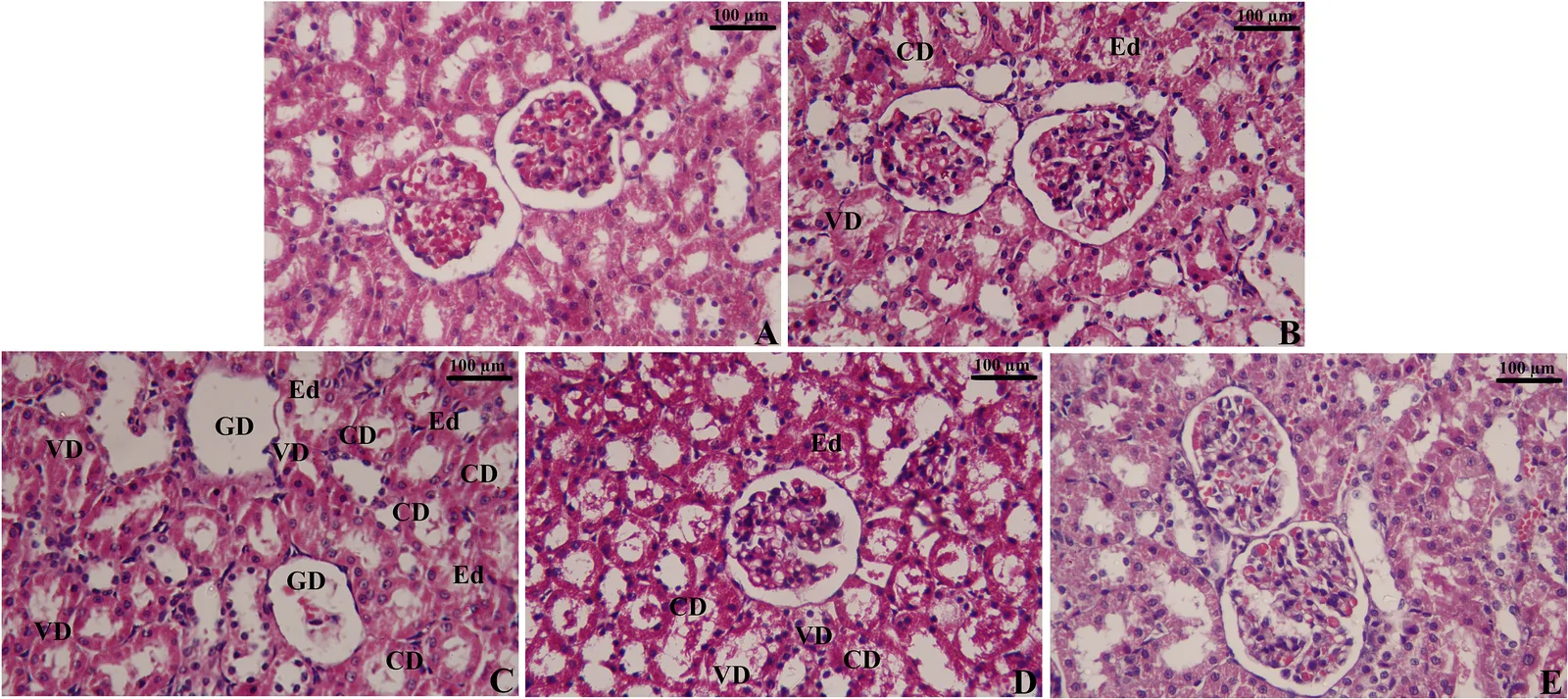
Shows renal tissue histological alterations in the groups under investigation. There are five different groups: **(a)** control, **(b)** sham, **(c)** CLP, **(d)** CLP-MSCs, and **(e)** CLP-MSCs + melatonin. 400×. GD, glomerular degeneration; VD, vacuolar degeneration; Ed, edema, and CD, cellular debris.

The glomerulus, proximal renal tubule, and distal renal tubule in the control (a) and sham (b) rats showed normal structure and no damage, according to histopathological features of the kidney sections (**Figure 9**). However, in addition to the infiltration of inflammatory cells in the interstitial tissue, the kidney tissue of the sepsis group (c) showed vacuolar degeneration, reduced Bowman’s gaps, and significant edema in the glomeruli. Along with cell desquamation, cellular debris was seen in the tubular lumen (**Figure 9**; **Table 2**). The normal histological architecture of renal glomeruli and tubules (**Figure 9**) indicates that renal tissue was protected by MSCs (d) and MSCs + melatonin (e) treatment.

**Table 2:** Effects of bone marrow–derived mesenchymal stem cells (MSCs) and MSCs-enhanced with melatonin on the semi-quantitative analysis severity of every pathological alteration in the renal tissues of CLP-induced septic rats.

Group	Control	Sham	CLP	CLP-MSCs	CLP-MSCs + melatonin
Semi-quantitative pathological score of renal tissue	0.49 ± 0.05	1.11 ± 0.45	3.36 ± 0.39^#^	1.94 ± 0.12^#$^	1.51 ± 0.14^#$^

Results of semi-quantitative pathological scores are presented as mean ± SD values of five fields. Statistical comparisons among groups were performed using one-way analysis of variance (ANOVA) followed by Tukey’s *post-hoc* multiple comparison test. ^#^Differs from the control group (*P* < 0.05). ^$^Differs from the septic rats (*P* < 0.05). ^@^Differs from the CLP-MSCs rats (*P* < 0.05).

## Discussion

Sepsis has been redefined as “life-threatening organ dysfunction resulting from a dysregulated host response to infection” (44). Since sepsis is a serious illness with a high morbidity and death rate, early detection and treatment are essential. So, this study investigated the protective effect of stem cells alone and stem cells enhanced with MEL against inflammation and oxidative stress induced by sepsis in an animal model.

This study demonstrates that preconditioning bone marrow–derived MSCs with MEL significantly enhances their therapeutic efficacy in a rat model of sepsis-induced acute kidney injury (AKI). The combined treatment (MSCs + MEL) proved superior to MSCs alone in restoring renal function, attenuating histopathological damage, and modulating the underlying molecular pathways of injury.

The pineal gland is principally responsible for the synthesis and release of MEL, a polypeptide that is generated from tryptophan. Its release exhibits considerable interindividual heterogeneity but notable intraindividual consistency (45). However, MEL exhibits a variety of effects in various bodily organs, including its ability to reduce inflammation, scavenge oxidants, and strengthen the immune system. MEL has emerged as a viable area of study for critically ill patients due to its extremely safe side-effect profile. MEL’s immunostimulatory, anti-inflammatory, and antioxidant qualities, as well as its impact on enhancing sleep architecture and cycling, have all been assessed when administered exogenously.

In numerous studies of sepsis models, MEL has been shown to enhance organ function and boost survival (46–50). MEL’s therapeutic benefits in these sepsis models stem from its influence on several pathways.

### Antioxidant properties

An oxidative imbalance with oxidant and antioxidant levels correlated with the severity of the illness is a hallmark of sepsis (46–48, 51). Free radicals can impair the glycocalyx’s ability to function and cause damage to proteins, lipids, and DNA (52, 53). The “free radical scavenging cascade” refers to the ability of MEL and its metabolites to scavenge ROS/RNS (54). When it comes to avoiding oxidative damage, MEL offers benefits over other antioxidants (55, 56). MEL and its metabolites (57–59) have strong antioxidant properties that shield mitochondria from oxidative damage because MEL reaches high concentrations within mitochondria (60). Additionally, MEL plays a role in the intra-mitochondrial SIRT3 pathway. SIRT3 is a class 3 histone deacetylase that guards against oxidative stress in mitochondria (61–63). MEL also promotes the production of additional antioxidant enzymes, such as glutathione reductase, glutathione peroxidase, y-glutamyl-cysteine synthetase, glucose-6 phosphate dehydrogenase, and catalase (64, 65). MEL has been shown to raise glutathione levels in experimental sepsis models (66). MEL has been shown to lessen hepatic necrosis in septic rats and to lower myeloperoxidase expression and malondialdehyde levels in the liver, brain, lung, and kidneys (49). MEL’s beneficial antioxidant properties have been reported in models of lipopolysaccharide (LPS)-induced liver failure and cecal ligation and puncture (CLP)-induced septic shock (46, 50, 66).

### Anti-inflammatory properties

An excessive pro-inflammatory reaction that causes organ dysfunction and eventually mortality is a hallmark of the early stages of sepsis. MEL has been shown to lower pro-inflammatory cytokine levels in a number of rodent models (46, 49, 67) and has strong anti-inflammatory and anti-apoptotic qualities (50, 65, 68–71). MEL reduced pulmonary inflammation in a rat model of LPS-induced acute lung injury; this was linked to an increase in the anti-inflammatory cytokine interleukin 10 (IL-10) and a decrease in the expression of nuclear factor kappa-β p65 (NF-κB p65) and tumor necrosis factor-α (TNF-α) (72).

In a mouse model of LPS-induced sepsis, MEL dose-dependently decreased serum TNF-γ and interleukin-6 (IL-6) (73). In a mouse model of sepsis treated with intraperitoneal MEL, attenuation of the cytokine response was also shown, and MEL considerably increased the survival rate (50). MEL has been shown to shut off NF-κB expression in a number of *in-vitro* models (74, 75). MEL dose-dependently reduced the expression of NF-κB and altered the expression of IL-6 and IL-8 in a human umbilical vein endothelial cell (HUVEC-C) model of sepsis (76). These anti-inflammatory effects could be achieved by reducing oxidative stress, inhibiting NF-κB, preventing apoptosis (77–80), or modulating the toll-like receptor (TLR) inflammatory cascade (81).

### Inhibiting nitric oxide production

Inducible cytosolic and mitochondrial isoforms of nitric oxide synthase (mtNOS and iNOS) are expressed more during sepsis, which raises NO levels and causes mitochondrial damage (82–84). Melatonin suppresses both iNOS and mtNOS isoforms in experimental models of sepsis (55, 82, 85). By reducing NO levels, inhibition of iNOS prevents organ failure and mortality (85). Additionally, melatonin can boost the activity of complexes I and IV, improving the ETC and limiting the generation of ROS and RNS (82, 86). Furthermore, MEL inhibits the activation of multiple calcium-dependent enzymes, including mtNOS and iNOS (82), by downregulating NOS activity through calmodulin (87–89). The MEL metabolite N-acetyl-5-methoxy Kynurenamine (AMK) showed a greater capacity to bind calmodulin and lower nNOS expression (90).

### Immune-enhancing properties

Wu et al. showed that MEL reduces IL-1β, decreases polymorphonuclear infiltration, attenuates oxidative stress, and lowers NO levels in rats following CLP (46). In an LPS model of sepsis, MEL decreases the generation of IL-6 (48), suppressing NF-κB activation, preventing mitochondrial damage, and turning off inflammasome-dependent cytokine production (76). MEL’s receptors, MT1 and MT2, which are frequently expressed on MSCs, may potentially influence cell destiny through receptor-dependent pathways (91–93). Sepsis-related AKI is a particularly serious type with substantial morbidity and death. In a CLP model, Chen et al. (23) compared MEL-preconditioned MSCs with MSCs alone. They discovered that the combined therapy produced better anti-inflammatory, antioxidant, anti-apoptotic, and anti-fibrotic responses, as well as lower serum creatinine levels and less renal damage on histological examination. MEL preconditioning significantly increases the proliferation of MSCs *in vitro* and upregulates important survival and antioxidant proteins such as phosphorylated Erk1/2, Akt, SOD-1, and heme oxygenase-1 (HO-1) (23). Additionally, Saberi et al. (94) showed that MEL administration improved BMSC homing to the damaged kidney, decreased renal cell apoptosis, and lowered TNF-α, α-SMA, and TGF-β1 expression. In the end, this strategy successfully reduced renal fibrosis, enhanced tissue architecture, and strengthened the tubular basement membrane.

In the present study, the serum level of CRP in the sepsis group increased significantly when compared to the control group. CRP is the most sensitive measure of inflammation, which rises quickly in the serum when body tissue is damaged or infected (95–97). CRP, which is produced by the liver acute-phase reactive protein, is a component of the innate immune system. This cyclic homopentameric protein participates in the acute-phase response during sepsis. It binds lipopolysaccharides in gram-negative organisms and phosphoryl choline, which is a part of teichoic acids in gram-positive organisms. Apoptotic cells’ exposed chromatin, histones, ribonucleoproteins, and lysophosphatidylcholine can all be bound by CRP. It initiates the conventional complement pathway and triggers phagocytosis by interacting with complement component C1q and the crystallizable antibody fragment (Fc) receptors Fc-γRI and Fc-γRII (98–100). Although it has been observed in neurons, monocytes, lymphocytes, and atherosclerotic plaques, CRP expression is mostly found in the liver (101). TNF-α, IL-1β, and interleukin (IL)-6 all stimulate the formation of CRP (98–100).

However, the level of CRP in both MSCs and MSCs + MEL groups was significantly reduced when compared to the level of CRP in the septic group. When bone marrow–derived MSCs were used to treat chronic inflammation, the proinflammatory cytokines like CRP in the animal serum of the treated groups were significantly decreased; the immunosuppressive characteristics of MSCs could be used to explain the outcomes. B cells, T cells, dendritic cells (DCs), natural killer cells (NK), neutrophils, and macrophages are just a few of the immune cell types that MSCs can interact with (102). It was demonstrated that MSC-regulated immunosuppression is induced by interaction mechanisms based on intercellular contact that cooperate with the release of soluble immune factors (103).

The data showed a significant increase in the serum levels of urea, creatinine, and KIM-1 in the septic group compared to the control group. These results are supported by previous studies on sepsis in rats (32, 104, 105). One well-known indicator of kidney damage is KIM-1, a transmembrane protein found in damaged proximal RTECs (106). Because local inflammatory mediators change renal microcirculation, affect kidney function, and cause tubular cell destruction and acute tubular necrosis, elevated BUN and creatinine in sepsis may be explained (107). Our data in MSCs and MSCs + MEL groups revealed a significant decrease in the levels of urea, creatinine, and Kim-1. A previous study reported that MSC treatment reduced the expression of KIM-1 in the kidney tissues of septic rats (105).

Moreover, there was a significant increase in the levels of oxidative stress markers (MDA, MPO, and NO) and a significant decrease in the activity of antioxidant markers (GSH, SOD, CAT, GPx, and GR) in the septic group when compared to the control group. SOD plays its antioxidant effects by transforming the toxic superoxide ion into a less toxic compound, which is H_2_O_2_, whereas the CAT enzyme breaks it into water and oxygen (108). Glutathione (GSH) is a very important antioxidant in the cells; it is found in both reduced (GSH) and oxidized (GSSG) forms. By the aid of the glutathione reductase enzyme, GSH can be restored from GSSG (109). GPx is found in cellular membranes and helps in the reduction of H_2_O_2_ by converting GSH into GSSG. Our findings are consistent with prior research (32, 110). Notably, the MSCs or MSC + MEL-treated groups had a significant decrease in the levels of MDA and NO and a significant increase in the levels of GSH and antioxidant enzyme activity (SOD, CAT, GPx, and GR) when compared to the septic group. These results are supported by previous research using the same method of treatment (111–113).

Our data showed that the serum levels of inflammatory markers (IL-6, TNF-α, IL-1β, PGE2, NF-κB, and COX2) were significantly increased in septic groups as compared to their levels in the control group. The polypeptide IL-6 regulates inflammation, the immune system, and blood production in a number of ways. Although other cells may also create it, monocytes and lymphocytes are the main cells that produce IL-6 in response to infections or tissue damage. In the case of an infectious process, binding to monocyte and macrophage pathogen-recognition receptors (PRRs) stimulates the production of IL-6. Numerous pathways, including NF-κB, are activated as a result, and mRNA transcription rises, causing the release of inflammatory cytokines. Damage-associated molecular patterns, or DAMPs, are released when tissue is injured. DAMPs from injured or dying cells contain a number of chemicals that promote the synthesis of IL-6 (114). NF-κB is one of the most significant transcription factors that controls the expression of genes encoding for numerous pro-inflammatory cytokines, including IL-1β and IL-6, and is implicated in the pathogenesis of kidney injury and the expression of genes related to the sepsis-induced inflammatory response (115). NF-κB inhibition inhibits intravascular coagulation, stops microvascular endothelial leakage, and lowers the expression of several pro-inflammatory genes (116). Severe infections typically cause hyperactivity and the release of endogenous inflammatory mediators, which can result in cytokine storm, severe tissue and organ damage, and even multiple organ failure (117). TNF-α is the primary mediator that controls the cascade of events in sepsis; its level is elevated and is correlated with the higher mortality rate in patients with sepsis. TNF-α stimulates specific transmembrane TNF receptors, leading to immune cell activation and the production of an array of downstream inflammatory mediators, which cause oxidative DNA damage. IL-1 is released by activated macrophages mostly in timely and functional manners like TNF-α (118).

Pro-inflammatory cytokine levels (IL-1b and TNF-α) were significantly reduced in septic rats treated with MSCs or MSCs + MEL when compared to the septic group, which supports earlier studies using the same treatment in different injury models (111, 119). A number of variables that regulate inflammatory status, including NO, PGE2 (prostaglandin E2), and TSG-6 (TNF alpha induced protein 6), were reduced by MSCs; this is in concordance with Pugazhenthi et al., 2008. Notably, MEL preconditioning for MSCs significantly reduced the amount of pro-inflammatory cytokines generated in the septic rats as compared to MSCs, thereby suggesting that MEL has enhanced the anti-inflammatory activity of MSCs. One of the most important target molecules for the control of inflammation is the transcription factor nuclear factor-kappa B. When activated, this protein complex moves into the nucleus and increases cytokine production (120). Our findings demonstrated significant reductions in NF-κB levels following MSCs or MSCs + MEL treatment, which could account for the anti-inflammatory effects of combined MEL and MSCs therapy. These results are consistent with earlier research (121–123). It has previously been demonstrated that inhibiting the active NF-κB pathway reduces the production of inflammatory genes (123). MEL treatment of septic rats was found to significantly trigger cyclooxygenase-2, which has anti-inflammatory effects (111).

The serum levels of proapoptotic markers (Bax and caspase 3) were significantly higher in the septic group than in the control group; also, the level of antiapoptotic marker Bcl-2 was reduced significantly. These results are supported by other studies that treated the same injury (124, 125). Most cells have caspases in an inactive proenzyme form, and once they are activated, they can frequently activate additional procaspases, which starts a protease cascade. Additionally, some procaspases have the ability to aggregate and autoactivate. Rapid cell death results from this proteolytic cascade, which increases the apoptotic signaling pathway by allowing one caspase to activate additional caspases. Once caspases are first activated, there appears to be an irreversible commitment to cell death. Caspases have proteolytic activity and can cleave proteins at aspartic acid residues (126).

Members of the Bcl-2 protein family are responsible for the regulation and control of these apoptotic mitochondrial processes (127). The Bcl-2 protein family is crucially regulated by the tumor suppressor protein p53. The Bcl-2 protein family, which can be either pro- or antiapoptotic, controls the permeability of the mitochondrial membrane. The Bcl-2 family has a total of 25 genes identified. Pro-apoptotic proteins include Bcl-10, Bax, Bak, Bid, Bad, Bim, Bik, and Blk, while anti-apoptotic proteins include Bcl-2, Bcl-x, Bcl-XL, Bcl-XS, Bcl-w, and BAG. Because they have the ability to decide whether a cell commits to apoptosis or aborts the process, these proteins are very important. The Bcl-2 protein family is believed to primarily regulate cytochrome c release from the mitochondria by modifying the permeability of the mitochondrial membrane (126).

While in both MSCs and MSCs + MEL groups the levels of Bax and Caspase 3 were significantly decreased compared to their levels in the septic group, the level of Bcl-2 was also significantly increased in both MSCs and MSCs + MEL groups compared to its level in the septic group, and this is supported by earlier studies (124, 128, 129).

In the current study, we explored the gene expression of iNOS, Caspase 3, and MAPK 9. It was found that in the septic group, the expression of these genes was significantly increased compared to that of the control group. These results were supported by other studies (130–133). Gram-negative bacteria’s cell wall component LPS binds to TLR4 to produce the CD14-LPS complex, which initiates the inflammatory signal. NF-κB is a pleiotropic transcription factor implicated in inflammation that is activated by the inflammatory signal triggered by adaptor proteins (134). Following the active NF-κB complex’s translocation to the nucleus, iNOS transcription is stimulated by the p65-p50 complex. NO generation is also triggered by other inflammatory cytokines, including TNF-α and IL-1β, which are released from infected cells (135). In order to control the release of pro-inflammatory cytokines and mediators, the MAPK and NF-κB/IκBα signaling pathways must be activated (136). It has been established that MAPKs are NF-κB upstream activators and play a crucial role in controlling NF-κB activation, which is linked to sepsis-related inflammation and apoptosis. According to a number of studies, p38 MAPK is crucial for NF-κB activation and migration to the nucleus, which in turn activates NF-κB activity and increases the expression of pro-inflammatory mediators, including TNF-α and IL-1β (137, 138). On the other hand, the expression of the inflammatory genes (iNOS, Caspase 3, and MAPK 9) was significantly reduced in MSC and MSC + MEL groups compared to those of septic groups, which is in concordance with the previous studies (111, 139). These results agree with the previous finding when we measured the serum levels of NO and Caspase 3 using the ELISA technique. Our data showed that MSCs + MEL presented an additional merit in alleviating CLP-induced sepsis, likely due to its anti-inflammatory, antioxidant, and anti-apoptotic potentials.

Histopathological analysis of kidney tissue in the septic group revealed renal tubular injury, apoptotic cells, glomerular capillary dilatation, renal interstitial edema, and the deformation and shedding of renal tubular epithelial cells. Furthermore, elevated levels of serum BUN, TNF-α, IL-1, and NF-κB have been documented (140, 141). Our study’s findings are in line with the conducted study in this regard. In addition to the improved biochemical parameters, an improvement in the histopathological picture of kidney sections and immunohistochemistry analysis of NF-κB and TNF-α was shown in MSC and MSC + MEL-treated animals compared to the septic group in accordance with (139, 142). In light of the study’s findings, elevated NF-B levels and histopathological reactions in the damaged kidney tissues demonstrate that an inflammatory scene forms at the tissue level. Inflammation is thought to be the cause of the glomerular congestion and shrinkage, distal tubule degeneration, mononuclear cell infiltration, cellular debris, and intense proteinous accumulation in the tubules that were discovered to be considerably higher in the septic group as compared to the control group using HE staining in accordance (143).

## Conclusion

This study demonstrates the therapeutic potential of MSCs preconditioned with MEL in mitigating sepsis-induced AKI in a rat model of CLP. The combined treatment (MSCs + MEL) exhibited superior renoprotective effects compared to MSCs alone, significantly reducing oxidative stress, inflammation, and apoptosis while enhancing antioxidant defenses and promoting tissue repair. Mechanistically, the synergistic action of MSCs and MEL attenuated NF-κB and TNF-α pathways, downregulated pro-inflammatory cytokines (IL-1β, IL-6, COX-2), and modulated apoptotic markers (Bax, Caspase-3, Bcl-2). Histopathological and immunohistochemical analyses confirmed the restoration of renal architecture and reduced inflammatory infiltration in treated groups. These findings highlight MEL’s role in enhancing MSC efficacy by improving their paracrine activity and survival, offering a promising combinatorial strategy for sepsis-associated AKI. Further clinical studies are warranted to validate these results and optimize dosing regimens for translational applications.

## Data Availability

The original contributions presented in the study are included in the article/supplementary material. Further inquiries can be directed to the corresponding author.
